# Autophagy-related gene model as a novel risk factor for schizophrenia

**DOI:** 10.1038/s41398-024-02767-5

**Published:** 2024-02-13

**Authors:** Yunfei Tan, Junpeng Zhu, Kenji Hashimoto

**Affiliations:** 1 Center for Rehabilitation Medicine, Department of Psychiatry, Zhejiang Provincial People’s Hospital (Affiliated People’s Hospital), Hangzhou Medical College, 310014 Hangzhou, Zhejiang China; 2 Division of Clinical Neuroscience, Chiba University Center for Forensic Mental Health, Chiba, 260-8670 Japan

**Keywords:** Clinical genetics, Schizophrenia

## Abstract

Autophagy, a cellular process where cells degrade and recycle their own components, has garnered attention for its potential role in psychiatric disorders, including schizophrenia (SCZ). This study aimed to construct and validate a new autophagy-related gene (ARG) risk model for SCZ. First, we analyzed differential expressions in the GSE38484 training set, identifying 4,754 differentially expressed genes (DEGs) between SCZ and control groups. Using the Human Autophagy Database (HADb) database, we cataloged 232 ARGs and pinpointed 80 autophagy-related DEGs (AR-DEGs) after intersecting them with DEGs. Subsequent analyses, including metascape gene annotation, pathway and process enrichment, and protein-protein interaction enrichment, were performed on the 80 AR-DEGs to delve deeper into their biological roles and associated molecular pathways. From this, we identified 34 candidate risk AR-DEGs (RAR-DEGs) and honed this list to final RAR-DEGs via a constructed and optimized logistic regression model. These genes include *VAMP7, PTEN, WIPI2, PARP1, DNAJB9, SH3GLB1, ATF4, EIF4G1, EGFR, CDKN1A, CFLAR, FAS, BCL2L1* and *BNIP3*. Using these findings, we crafted a nomogram to predict SCZ risk for individual samples. In summary, our study offers deeper insights into SCZ’s molecular pathogenesis and paves the way for innovative approaches in risk prediction, gene-targeted diagnosis, and community-based SCZ treatments.

## Introduction

Schizophrenia (SCZ) is a chronic mental disorder characterized by abnormalities in sensory, perceptual, emotional, and behavioral functions. Patients often struggle to differentiate between reality and imagination, exhibit delayed reactions, and may show either withdrawn or exaggerated behaviors. In severe cases, they face challenges with normal social interactions [1, 2]. The onset of the disease typically occurs in youth or early adulthood. While there’s increasing evidence pointing to hereditary factors, abnormal brain structures, complications during pregnancy, and environmental influences, the precise pathogenesis and etiology remain elusive. Although a definitive cure for schizophrenia has not yet been discovered, appropriate treatments can effectively manage its symptoms [3–6].

Autophagy is a cellular process in which cytoplasmic proteins or organelles are encapsulated in vesicles. These vesicles then fuse with lysosomes to form autolysosomes, breaking down their contents to support the cell’s metabolic needs and to renew certain organelles [6]. Autophagy has a pivotal role in safeguarding the body during the progression of many diseases [7]. Yet, in the context of tumor formation and development, autophagy exhibits a dual effect [8]. It can trigger programmed cell death, reducing the chance of DNA mutations, thus playing an anti-tumor role [9]. However, in less favorable cellular environment, autophagy may offer a lifeline for tumor cells, supporting their growth and replication [10]. Consequently, whether autophagy ultimately hinders or facilitates tumor progression could be intimately linked to the surrounding cellular environment and the level of autophagy at its onset [11].

Autophagy-related genes (ARGs) are implicated in the pathophysiology of SCZ [12–16]. A study of postmortem brain samples has revealed reduced levels of beclin1 in the hippocampus of SCZ patients [17]. Moreover, alterations in the gene expressions of mTOR (or FOXO) pathway-related ARGs were observed in blood samples from SCZ patients compared to healthy controls, with further significant changes following a 4-week treatment with olanzapine [18, 19]. Antipsychotics such as phenothiazines have been noted to regulate autophagy, contributing to their therapeutic effects in SCZ patients [20]. A recent meta-analysis highlighted that phenothiazine-like antipsychotics, including chlorpromazine, fluphenazine, methotrimeprazine, perphenazine, prochlorperazine, promethazine, thioridazine, and trifluoperazine, can modulate autophagy [21]. Overall, the role of autophagy in SCZ is multifaceted, encompassing neuronal homeostasis, disease pathophysiology, and symptom modulation, with its regulation being mediated through specific genes and pathways, underscoring the complexity and significance of this process in SCZ.

Considering the evidence, we hypothesized that ARGs could be diagnostically relevant for SCZ and may influence its onset and progression. To explore this hypothesis, our study focused on the molecular biological roles of ARGs in SCZ. We aimed to develop and validate an ARG-based risk model for the disorder. For this purpose, we utilized data from the Gene Expression Omnibus (GEO) database, which is curated by the National Center for Biotechnology Information (NCBI).

## Materials and methods

### Sample data collection and sorting

The GEO database by NCBI houses a diverse array of gene expression data, including second-generation sequencing, chip sequencing, and single-cell sequencing data [22]. For our study, we chose and downloaded two datasets from the GEO database based on specific criteria: they had to include human samples with SCZ and control groups, and the complete raw data must be available. We excluded any datasets with incomplete metadata, post-mortem samples, or those affected by confounding treatment effects. We analyzed two datasets: GSE38484, which includes 106 SCZ and 96 control samples, and GSE38481, containing 15 SCZ and 22 control samples. Probe IDs from these datasets were mapped to gene symbols using the platform’s annotation file for accurate gene identification. We transformed each gene’s expression value using log^2^ to stabilize variance and enhance the interpretability of both low and high expression values, a standard practice in bioinformatics. This transformation produced the final gene expression matrix [23]. For all statistical analyses, we used R software (Version 4.0.2, R Foundation for Statistical Computing, Vienna, Austria), employing the “limma” package specifically for differential expression analysis.

### Screening of autophagy-related differentially expressed genes (AR-DEGs) for SCZ

Firstly, we designated GSE38484 as the training set and employed the “wilcox” function, using the R package “limma”, to calculate differences in gene expression between the SCZ and control groups, thereby identifying differential expression genes (DEGs) [24]. Subsequently, we retrieved the list of autophagy-related genes (ARGs) from the Human Autophagy Database (HADb) (http://www.autophagy.lu). We derived AR-DEGs by intersecting DEGs with the ARGs [25]. Furthermore, we utilized the R software package for visual representation of the results. An adjusted P-value of less than 0.05 was deemed statistically significant.

### Metascape gene list analysis for AR-DEGs

Metscape is an advanced gene function annotation analysis tool that empowers researchers to deploy comprehensive bioinformatics analyses on target genes or proteins, providing deeper insights into their molecular biological functions [26]. We input the AR-DEGs list into the Metscape database, choosing Homo sapiens as the species for gene annotation, pathway and process enrichment analysis, as well as protein-protein interaction (PPI) enrichment analysis [27]. We selected terms according to predefined criteria: P-values less than 0.01, a minimum occurrence of 3, and an enrichment factor exceeding 1.5. The enrichment factor is defined as the ratio of observed to expected counts. These selected terms were then categorized into clusters based on their similarity, as detailed in Table 1. For a more detailed analysis of the interconnections among these terms, we focused on a subset of enriched terms and represented them in a network diagram. In this diagram, terms were connected if their similarity exceeded 0.3. We utilized Cytoscape for visualization, where each node represents an enrichment term and is color-coded according to its cluster ID and P-value.

**Table 1:** Top 20 clusters with their representative enriched terms (one per cluster).

GO	Category	Description	Count	%	Log10(P)	Log10(q)
hsa04140	KEGG Pathway	Autophagy - animal	27	33.75	−43	−38.65
GO:0010506	GO Biological Processes	Regulation of autophagy	26	32.5	−30.08	−26.34
GO:0062197	GO Biological Processes	Cellular response to chemical stress	22	27.5	−25.87	−22.3
hsa05417	KEGG Pathway	Lipid and atherosclerosis	18	22.5	−21.6	−18.21
hsa05131	KEGG Pathway	Shigellosis	18	22.5	−20.5	−17.15
GO:0071453	GO Biological Processes	Cellular response to oxygen levels	15	18.75	−18.79	−15.64
GO:0071496	GO Biological Processes	Cellular response to external stimulus	18	22.5	−18.4	−15.28
GO:0010942	GO Biological Processes	Positive regulation of cell death	21	26.25	−17.24	−14.31
WP1772	WikiPathways	Apoptosis modulation and signaling	12	15	−16.82	−13.95
hsa04137	KEGG Pathway	Mitophagy - animal	11	13.75	−16.24	−13.44
WP3611	WikiPathways	Photodynamic therapy-induced AP-1 survival signaling	10	12.5	−15.96	−13.21
GO:2001233	GO Biological Processes	Regulation of apoptotic signaling pathway	17	21.25	−15.7	−12.99
hsa05215	KEGG Pathway	Prostate cancer	11	13.75	−14.75	−12.09
hsa04211	KEGG Pathway	Longevity regulating pathway	10	12.5	−13.4	−10.83
GO:2000377	GO Biological Processes	Regulation of reactive oxygen species metabolic process	11	13.75	−12.65	−10.14
WP4925	WikiPathways	Unfolded protein response	7	8.75	−12.64	−10.13
WP5087	WikiPathways	Malignant pleural mesothelioma	15	18.75	−12.21	−9.73
R-HSA-449147	Reactome Gene Sets	Signaling by Interleukins	15	18.75	−11.76	−9.32
hsa04068	KEGG Pathway	FoxO signaling pathway	10	12.5	−11.68	−9.24
GO:0071417	GO Biological Processes	Cellular response to organonitrogen compound	16	20	−11.45	−9.03

“Count” is the number of genes in the user-provided lists with membership in the given ontology term. “%“ is the percentage of all of the user-provided genes that are found in the given ontology term (only input genes with at least one ontology term annotation are included in the calculation). “Log10(*P*)” is the p-value in log base 10. “Log10(*q*)” is the multi-test adjusted *p*-value in log base 10.

### Construction of SCZ risk model based on risk AR-DEGs (RAR-DEGs)

Initially, we accessed the list of AR-DEGs and extracted their expression values based on the gene expression matrix of the training set, further distinguishing sample grouping information as either the SCZ or control group. Utilizing the R package “glmnet”, we executed the Least Absolute Shrinkage and Selection Operator (LASSO) regression analysis, setting the response type to binomial and identifying alpha identified as 1. By minimizing the binominal deviation criterion, the optimal λ (representing the number of candidate RAR-DEGs of SCZ) was determined through a 10-fold cross-validation that aimed to achieve the smallest cross-validation errors [28, 29]. Subsequently, a logistic regression model was constructed centered around the candidate RAR-DEGs. This model was optimized to derive the final RAR-DEGs. From the model’s risk calculation formula, we computed the SCZ risk score for each sample [30].

### Analysis and evaluation of nomogram based on risk model for SCZ

A nomogram serves as a dependable tool for quantifying risks associated with various diseases, enabling personalized predictions for a given sample’s disease risk [31]. We loaded both the risk model and the clinical information files, retaining only samples with the intersecting information. Leveraging R packages like “rms”, “rmda”, and “Hmisc”, and using RAR-DEGs expression values along with clinical information as independent variables and sample grouping as the dependent variable, we constructed a visual nomogram and accompanying calibration curve. Lastly, referencing the R packages “glmnet”, “pROC”, and “ggsci”, we plotted curves for each parameter variable via receiver operating characteristic (ROC) curve analysis and decision curve analysis (DCA) [32, 33]. this was done to validate the risk model’s and nomogram’s precision in predicting SCZ.

### Validation analysis of test set samples

To further validate the reliability of our SCZ risk model and the nomogram, both constructed using the GSE38484, we employed the GSE38481 dataset as a test set to visualize the expression values of the identified RAR-DEGs. We then performed calibration curve, ROC curve, and DCA analyses on the nomogram built from the risk model.

## Results

### Identification of AR-DEGs between SCZ and control groups

**Fig. 1:**
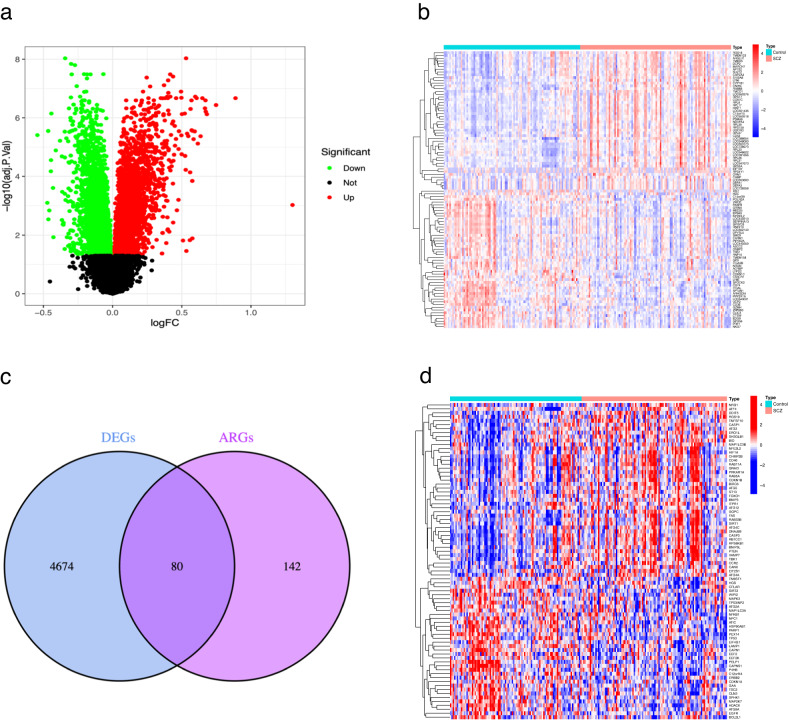
The DEGs expression between SCZ and control groups. The volcano plot (**a**) and heatmap (**b**) display the DEGs expression between the SCZ and control groups. The Venn diagram (**c**) illustrates the overlap of genes (AR-DEGs), while the heatmap (**d**) depicts the expression of AR-DEGs between the SCZ and control groups. Red dots or squares represent upregulated genes, while green dots or blue squares signify downregulated genes.

We combined the datasets GSE38484 and GSE38481 to produce two gene expression matrices, further detailed in supplementary documents S1 and S2. From the differential expression analysis of the GSE38484 training set, we identified 4,754 DEGs between the SCZ and the control groups: 2288 were up-regulated and 2466 were down-regulated (Fig. 1a). The top 50 most significantly up-regulated and down-regulated DEGs were visualized (Fig. 1b). Additionally, we sourced 232 ARGs from the HADb database, with 80 AR-DEGs identified upon intersecting with DEGs (Fig. 1c). Figure 1d depicts the differential expression of these AR-DEGs between the SCZ and control groups.

### Gene annotation and enrichment analysis on AR-DEGs

**Fig. 2:**
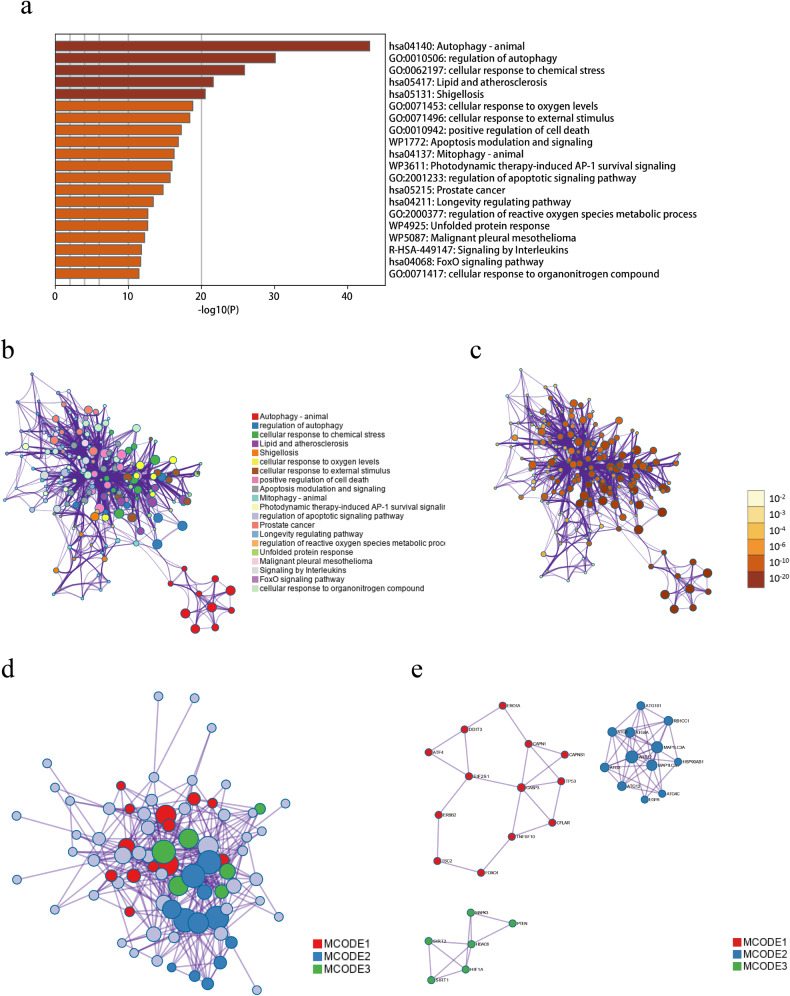
The input gene lists for SCZ. **a** A Bar graph illustrates enriched terms across the input gene lists, with colors indicating *P*-values. For the network of enriched terms: (**b**) it is colored by cluster ID, with nodes of the same cluster ID typically positioned near one another; (**c**) it is colored by *p*-value, indicating that terms with more genes usually have a more significant *p*-value. The protein-protein interaction (PPI) network is shown in (**d**), and MCODE components derived from the AR-DEGs list are presented in (**e**).

We conducted comprehensive metascape gene annotation, pathway and process enrichment analysis, and PPI enrichment analysis on the 80 AR-DEGs. Supplementary document S3 provides detailed annotations and enrichment information for these AR-DEGs. Figure 2a captures the functional or pathway enrichments of the AR-DEGs. The cluster ID and *P*-value were symbolized in Fig. 2b, c, respectively. Leveraging the list of 80 AR-DEGs, we formulated the PPI network (Fig. 2d) and MCODE component (Fig. 2e) using the STRING and BioGrid databases. Each MCODE component underwent pathway and process enrichment analysis, retaining the three highest *P*-values items as functional descriptions of the respective components (Table 2a, b).

**Table 2:** (a) The functional description of corresponding components of the three best-scoring terms by *P*-value. (b) The functional description of each MCODE component.

GO	Description	Log10(*P*)
(a)		
hsa04140	Autophagy - animal	−43.2
GO:0006914	Autophagy	−35
GO:0061919	Process utilizing autophagic mechanism	−35

MCODE	GO	Description	Log10(*P*)
(b)			
MCODE_1	hsa04210	Apoptosis	−15.8
MCODE_1	hsa05417	Lipid and atherosclerosis	−11.9
MCODE_1	GO:0043065	Positive regulation of apoptotic process	−10.9
MCODE_2	R-HSA-9612973	Autophagy	−24.4
MCODE_2	R-HSA-1632852	Macroautophagy	−21.8
MCODE_2	hsa04140	Autophagy - animal	−21.6
MCODE_3	GO:0034599	Cellular response to oxidative stress	−9.8
MCODE_3	GO:0034983	Peptidyl-lysine deacetylation	−9.6
MCODE_3	GO:0062197	Cellular response to chemical stress	−9.4

### Construction of SCZ risk model based on 14 RAR-DEGs

**Fig. 3:**
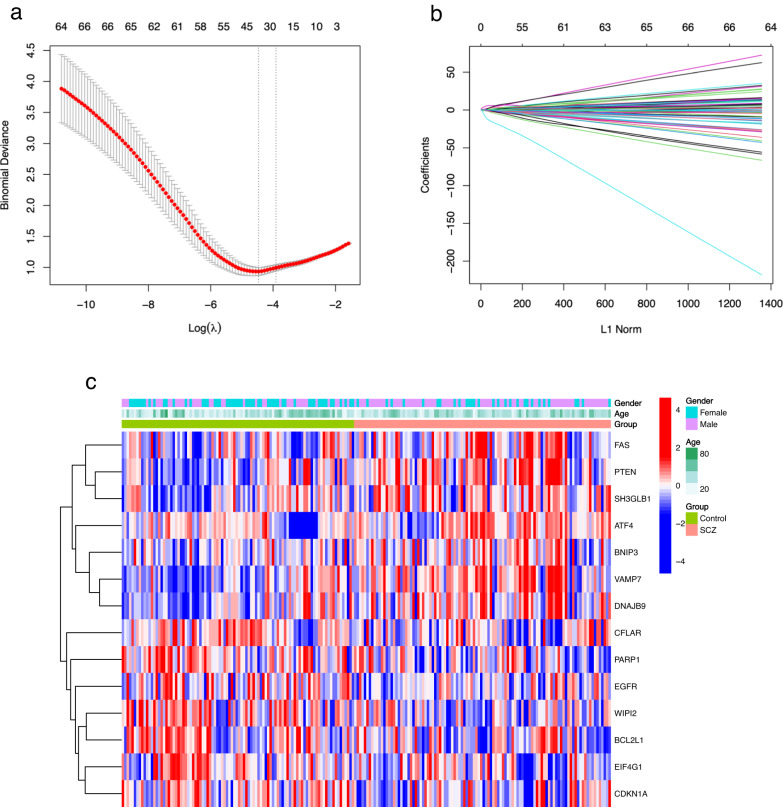
Identification of 14 RAR-DEGs in SCZ. A deviation curve (**a**) and a coefficient curve (**b**) highlight RAR-DEGs pinpointed through LASSO regression. **c** A heatmap showcases the expression of 14 RAR-DEGs across clinical phenotypes. Blue squares denote down-regulated genes, while red squares indicate up-regulated genes.

Utilizing lasso regression analysis combined with cross-validation, we pinpointed 34 candidate RAR-DEGs (Fig. 3a, b). Subsequently, we structured a logistic regression model, which, after optimization, revealed 14 RAR-DEGs, specifically: *VAMP7 (Vesicle-Associated Membrane Protein 7), PTEN (Phosphatase And Tensin Homolog), WIPI2 (WD Repeat Domain, Phosphoinositide Interacting 2), PARP1 (Poly(ADP-Ribose)Polymerase 1), DNAJB9 (DnaJ Heat Shock Protein Family Member B9), SH3GLB1 (SH3 Domain Containing GRB2 Like, Endophilin B1), ATF4 (Activating Transcription Factor 4), EIF4G1 (Eukaryotic Translation Initiation Factor 4 Gamma 1), EGFR (Epidermal Growth Factor Receptor), CDKN1A (Cyclin Dependent Kinase Inhibitor 1A), CFLAR (CASP8 And FADD Like Apoptosis Regulator), FAS (Fas Cell Surface Death Receptor), BCL2L1 (BCL2 Like 1)* and *BNIP3 (BCL2 Interacting Protein 3)* (Table 3). Figure 3c illustrates the differential expressions of these RAR-DEGs across various clinical phenotypes, including age, gender, and group. Additionally, the supplementary document S4 (Risk matrix.xls) displayed the expression levels of each RAR-DEGs in individual sample along with the associated SCZ risk scores.

**Table 3:** Fourteen RAR-DEGs for SCZ.

Genes	Full name of genes
*VAMP7*	*Vesicle-Associated Membrane Protein 7*
*PTEN*	*Phosphatase And Tensin Homolog*
*WIPI2*	*WD Repeat Domain, Phosphoinositide Interacting 2*
*PARP1*	*Poly(ADP-Ribose)Polymerase 1*
*DNAJB9*	*DnaJ Heat Shock Protein Family Member B9*
*SH3GLB1*	*SH3 Domain Containing GRB2 Like, Endophilin B1*
*ATF4*	*Activating Transcription Factor 4*
*EIF4G1*	*Eukaryotic Translation Initiation Factor 4 Gamma 1*
*EGFR*	*Epidermal Growth Factor Receptor*
*CFLAR*	*CASP8 And FADD Like Apoptosis Regulator*
*FAS*	*Fas Cell Surface Death Receptor*
*BCL2L1*	*BCL2 Like 1*
*BNIP3*	*BCL2 Interacting Protein 3*

### Construction of nomogram and internal validation of SCZ risk model

**Fig. 4:**
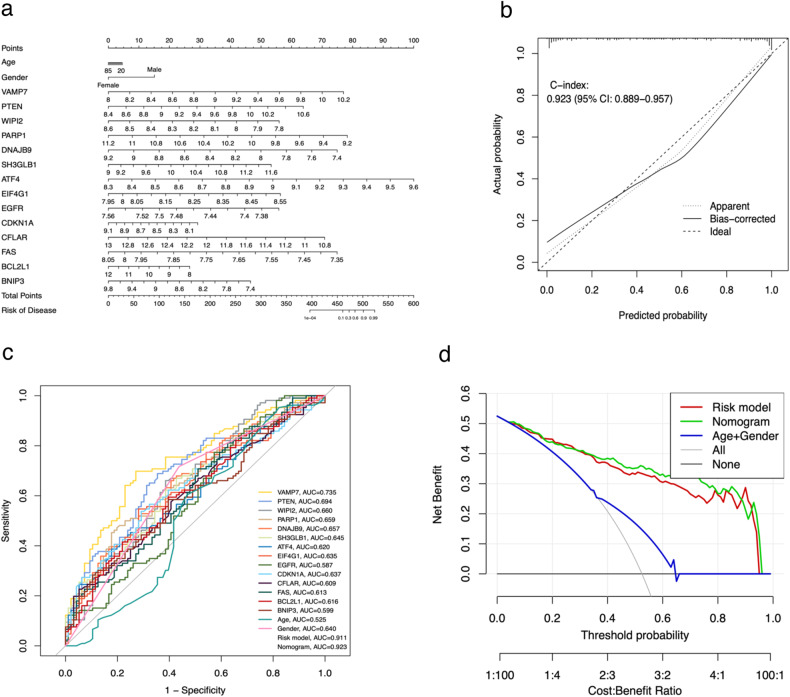
A nomogram, calibration curves, ROC and DCA curves. **a** A nomogram was developed using 14 RAR-DEGs and clinical characteristics (age and gender). To calculate the total score for each sample, draw a vertical line from the predictor’s scale to the score scale and then sum the resulting scores. **b** Calibration curves of the nomogram. The X-axis displays the nomogram’s predicted probability, whereas the *Y*-axis shows the actual SCZ probability. A perfect prediction would align with a 45° diagonal line. The dotted line represents the entire cohort (*n* = 202), while the solid line has been bias-corrected through bootstrapping (*B* = 1000 repetitions). **c** ROC curves for the risk model, nomogram, clinical features, and RAR-DEGs. **d** DCA curves for the risk model, nomogram and clinical features.

Using the SCZ risk model as a foundation, we developed a nomogram to individually estimate the risk of SCZ for a specific sample. As depicted in Fig. 4a, for any given sample, both clinical phenotype (age and gender) and the expression values of RAR-DEGs corresponded to specific point scale values. These points were then summed to determine a total point score, which corresponds to the SCZ disease risk score value, representing the individual’s risk for SCZ. The nomogram’s calibration curve, as seen in Fig. 4b, exhibits a close alignment between predicted and observed outcomes, suggesting that the nomogram’s SCZ probability predictions are largely consistent with the actual occurrences. Furthermore, the ROC curve indicated that both the risk model and the nomogram, based on 14 RAR-DEGs, have AUC values of 0.911 and 0.923, respectively (Fig. 4c). The DCA curve, shown in Fig. 4d, highlights that the curves of the risk model and the nomogram deviate significantly from the “ALL” curve. This demonstrated that our constructed risk model and nomogram offer high predictive accuracy for SCZ risk in samples, outperforming predictions based solely on other clinical phenotypes.

### External validation of SCZ risk model

**Fig. 5:**
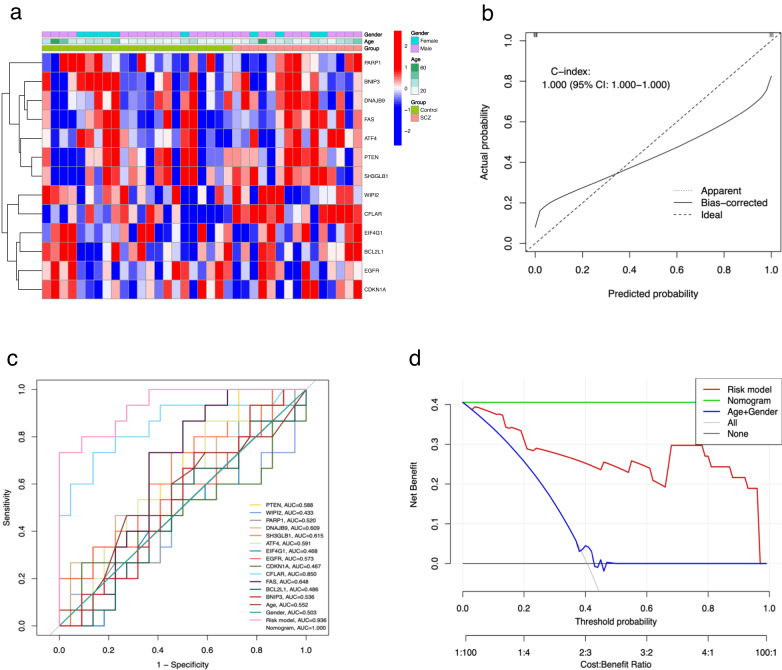
External validation of the SCZ risk model using the test set GSE38481. **a** A heatmap displaying RAR-DEGs expression across clinical phenotypes. Blue squares indicate down-regulated genes, while red squares denote up-regulated genes. **b** Calibration curves of the nomogram. The *X*-axis displays the nomogram’s predicted probability, and the *Y*-axis shows the actual SCZ probability. A perfect prediction would align with a 45° diagonal line. The dotted line corresponds to the entire queue (*n* = 37), and the solid line has been adjusted for bias using bootstrapping (*B* = 1000 repetitions). **c** ROC curves comparing the risk model, nomogram, clinical features, and RAR-DEGs. **d** DCA curves for the risk model, nomogram and clinical features.

We used the dataset GSE38481 as the test set to validate the differential expression of these RAR-DEGs between the SCZ and control groups (Fig. 5a). Moreover, using data from the test set, we plotted calibration curves (Fig. 5b), ROC curves (Fig. 5c), and DCA curves (Fig. 5d) for the established risk model. These visualizations further underscored the high accuracy and reliability of our risk model and the accompanying nomogram in predicting SCZ risk.

## Discussion

The etiology of SCZ remains unclear, but individual psychological vulnerabilities combined with external social stressors may contribute to the disease’s onset and progression. These factors might trigger the disease through their interplay with internal biological factors, with the pathogenesis of different patients potentially leaning more heavily on one factor over other’s [34, 35]. A substantial body of evidence suggests that genetic predispositions are significant risk factors for the development of SCZ [36]. It is widely accepted that SCZ is a multifaceted psychiatric disorder influenced by multiple genes, suggesting a polygenic inheritance pattern [37]. Specific genes linked to SCZ have been identified on chromosomes 6, 8, and 13. Studies using twin pairs have shown that identical twins exhibit a notably higher prevalence of SCZ compared to fraternal twins. We also find that ARG is associated with symptoms of SCZ. Dysfunction in neuronal autophagy, known to be involved in ARG regulation, are increasingly associated with the positive symptoms of SCZ [12, 17]. Furthermore, a comprehensive proteomic analysis revealed a significant enrichment of ARG-related signaling pathways in SCZ cases [17, 38]. This body of evidence suggests a notable association between ARG and the cognitive symptoms observed in SCZ. Furthermore, studies on adopted children revealed that when one biological parent has SCZ, children who were adopted into unaffected families still exhibited a substantially higher risk of developing SCZ compared to the general population [39]. Nonetheless, the precise genetic blueprint of SCZ remains to be fully deciphered.

In this study, we discovered that the primary functions of the investigated elements revolve around the regulation of autophagy, cellular responses to chemical stress, oxygen levels, external stimuli and organonitrogen compounds. These elements also play a significant role in promoting cell death, participating in the apoptotic signaling pathway, and managing processes specific to reactivate oxygen species. Additionally, we observed that their pathways and associated processes are notably enriched in several key areas. These include autophagy, shigellosis, prostate cancer, malignant pleural mesothelioma, lipid-related atherosclerosis, mitophagy, the unfolded protein response, apoptosis modulation and signaling, photodynamic therapy-induced AP-1 survival signaling, the longevity-regulating pathway, interleukin signaling, and the FoxO signaling pathway.

In our study, we identified 34 RAR-DEGs. Using a logistic regression model, we narrowed this down to a critical set of 14 RAR-DEGs (Table 3). Previous studies have highlighted the regulatory significance of certain RAR-DEGs in SCZ. For instance, Hong et al. [40] demonstrated that mice lacking *Parp1* exhibited SCZ-like behavioral symptoms, such as anxiety, depression, social interaction deficits, and cognitive impairment, suggesting the role of PARP1 in SCZ-associated behavioral abnormalities in mice. Another gene of interest, ATF4, is located on chromosome 22q13, a region associated with SCZ. Qu et al. [41] identified 18 single nucleotide polymorphisms (SNPs) in the *ATF4* locus; notably, the allele distribution of two SNPs was significantly associated with male SCZ patients. This indicates a possible link between the *ATF4* gene and SCZ susceptibility, potentially with sex-specific differences. Further, a study by Wang et al. [42] revealed that the antipsychotic drug paliperidone could reverse the reduction in PP2A and PTEN levels observed in neurons of prefrontal cortex induced by MK-801. This suggests that paliperidone may mitigate MK-801-induced neuronal damage through PP801A/PTEN pathway. In addition, several studies based on animal models align with our conclusions. Recent studies have illuminated the complex roles of specific genes in regulating brain functions and their implications in SCZ. PTEN mutations disrupt the brain’s excitatory/inhibitory balance, influencing traits related to intelligence, cognitive function, and SCZ. The PP2A/PTEN pathway has been identified as crucial in mitigating neuronal damage, offering therapeutic potential. CDKN1A’s upregulation following antipsychotic exposure during pregnancy links to altered apoptotic gene expressions, potentially affecting SCZ onset. One report showed SCZ-like phenotypes in mice lacking PARP1 gene. Lastly, ATF4 is pivotal in modulating neuronal excitability and receptor functionality, particularly in conditions like SCZ, by regulating GABA-B receptor trafficking. These findings contribute significantly to understanding SCZ’s genetic and molecular basis.

ARGs are essential in the biological mechanisms associated with SCZ [12–16]. First, autophagy is a cellular process that maintains homeostasis by eliminating damaged or unnecessary cellular components [43]. Alterations in this system might play a role in the pathophysiology of SCZ. Second, dysfunctions in ARGs could disrupt autophagy’s protein quality control, potentially leading to neuronal abnormalities seen in SCZ. Third, modifications in ARGs can affect synaptic plasticity and neurodevelopment, which are often linked to the onset and progression of SCZ. Additionally, aberrations in autophagic processes due to ARGs might contribute to the oxidative stress observed in the brains of SCZ patients [44–46]. Fourth, since autophagy influences neurotransmitter systems [47], alterations in ARGs could relate to the symptoms of SCZ patients. Fifth, genetic variations in ARGs, such polymorphisms or mutations, may disrupt normal autophagy increase susceptibility to SCZ [12]. Lastly, irregularities in autophagy may intensify neuroinflammatory processes [48, 49], aggravating the disease’s progression. Altogether, ARGs in SCZ are involved in several key processes mentioned above. Understanding these roles is crucial in deciphering SCZ’s complex pathology and could open doors to new therapeutic approaches.

This study has the following limitations that warrant further investigation in future research. Notably, the absence of a subgroup analysis for different clinical subtypes of SCZ and the failure to consider non-genetic factors that might affect our findings are significant drawbacks. The current study does not clarify the relationship between the expression of ARGs and various demographic and clinical characteristics of SCZ patients, such as sex, age of onset, illness of duration, or specific symptoms like positive and negative symptoms or cognitive impairments. Additionally, to corroborate our findings, acquiring a larger dataset and more comprehensive clinical information is essential, followed by vigorous empirical validation. Lastly, future research using rodent models featuring either knock-out or overexpression of ARGs will be instrumental in deepening our understanding of the role of ARGs in SCZ.

In conclusion, this study significantly enhances our understanding of the molecular mechanisms of ARGs in SCZ. Future research in SCZ would focus on identifying and understanding specific ARGs, exploring genetic factors influencing ARG pathways, developing targeted pharmacological treatments, and identifying biomarkers for early detection and progression monitoring of the disease.

### Supplementary information

Supplementary documents S1

Supplementary documents S2

Supplementary documents S3

Supplementary documents S4

## Data Availability

The dataset used and/or analyzed during this study can be made available upon request by contacting the corresponding author, Dr. Yunfei Tan.
